# Cuprotosis clusters predict prognosis and immunotherapy response in low-grade glioma

**DOI:** 10.1007/s10495-023-01880-y

**Published:** 2023-09-15

**Authors:** Wenjun Zhu, Ziqi Chen, Min Fu, Qianxia Li, Xin Chen, Xiaoyu Li, Na Luo, Wenhua Tang, Feng Yang, Yiling Zhang, Yuanyuan Zhang, Xiaohong Peng, Guangyuan Hu

**Affiliations:** 1 Department of Oncology, Tongji Hospital, Tongji Medical College, Huazhong University of Science and Technology, Wuhan, 430030 China; 2 Department of Oncology, Hubei Cancer Hospital, Wuhan, 430030 China; 3 Department of Oncology and Southwest Cancer Center, Southwest Hospital, Third Military Medical University (Army Medical University), Chongqing, 400038 China; 4 Department of Radiology, The First Affiliated Hospital, Zhejiang University School of Medicine, Hangzhou, China

**Keywords:** Bioinformatics, Low-grade glioma, Cuprotosis, Cluster, Prognosis, TME, Immunotherapy

## Abstract

Cuprotosis, an emerging mode of cell death, has recently caught the attention of researchers worldwide. However, its impact on low-grade glioma (LGG) patients has not been fully explored. To gain a deeper insight into the relationship between cuprotosis and LGG patients’ prognosis, we conducted this study in which LGG patients were divided into two clusters based on the expression of 18 cuprotosis-related genes. We found that LGG patients in cluster A had better prognosis than those in cluster B. The two clusters also differed in terms of immune cell infiltration and biological functions. Moreover, we identified differentially expressed genes (DEGs) between the two clusters and developed a cuprotosis-related prognostic signature through the least absolute shrinkage and selection operator (LASSO) analysis in the TCGA training cohort. This signature divided LGG patients into high- and low-risk groups, with the high-risk group having significantly shorter overall survival (OS) time than the low-risk group. Its predictive reliability for prognosis in LGG patients was confirmed by the TCGA internal validation cohort, CGGA325 cohort and CGGA693 cohort. Additionally, a nomogram was used to predict the 1-, 3-, and 5-year OS rates of each patient. The analysis of immune checkpoints and tumor mutation burden (TMB) has revealed that individuals belonging to high-risk groups have a greater chance of benefiting from immunotherapy. Functional experiments confirmed that interfering with the signature gene TNFRSF11B inhibited LGG cell proliferation and migration. Overall, this study shed light on the importance of cuprotosis in LGG patient prognosis. The cuprotosis-related prognostic signature is a reliable predictor for patient outcomes and immunotherapeutic response and can help to develop new therapies for LGG.

**Supplementary Information:**

The online version contains supplementary material available at 10.1007/s10495-023-01880-y.

## Introduction

LGG is a common intracranial primary malignant tumor, accounting for approximately 20% of primary tumors in the brain, with a median survival of between 4.7 and 9.8 years. LGG mainly includes diffuse astrocytoma, pilomyxoid astrocytoma, oligodendroglioma, oligoastrocytomas, and ganglioglioma, etc. [1, 2]. The primary treatment strategy for LGG involves surgical intervention, which is often followed by radiotherapy for patients with high-risk factors [3]. However, despite these attempts at treatment, a majority of LGG patients do not respond well and often experience a high recurrence rate. As a result, ongoing research is focusing on developing new treatment options to improve outcomes for LGG patients.

Recently, numerous studies have highlighted the correlation between genetic markers and the OS of LGG patients. For instance, 1p-19q deletion is considered a highly reliable indicator of response to chemotherapy and survival, and can also be used for diagnosing oligodendroglioma [4]. Additionally, mutations in isocitrate dehydrogenase 1 and 2 (IDH1 and IDH2) have been linked to better survival outcomes and higher rates of response to the drug temozolomide in LGG [5]. Furthermore, thanks to recent advances in genetic profiling, it is now possible to distinguish between different outcomes in LGG patients based on their genetic makeup [6, 7]. By identifying key biomarkers that can predict patient-specific OS, clinicians can optimize treatment plans and improve survival rates. A recent study has uncovered a novel form of cell death, known as cuprotosis, which has been linked to copper toxicity and closely associated with cellular mitochondrial respiration [8]. The excessive accumulation of copper within cells can be transported into the mitochondria by ion carriers, which results in a direct binding with lipid acylated components in the mitochondrial respiratory tricarboxylic acid cycle. This interaction, in turn, triggers an aggregation of lipid acylated proteins and a loss of iron-sulfur cluster proteins, initiating protein toxicity stress and eventually leading to cell death [8, 9]. In addition, researches have shown that genes related to cuprotosis play a critical role in the progression of tumors. For instance, the FDX1 gene affects the prognosis of patients with lung adenocarcinoma via its participation in fatty acid oxidation and glucose and amino acid metabolism [10]. Meanwhile, upregulating PDHA1 gene expression can inhibit the Warburg effect and enhance the mitochondrial-mediated apoptotic pathway in hepatocellular carcinoma cells [11]. In central nervous system (CNS) tumors, the cuprotosis-related gene dihydrolipoamide dehydrogenase (DLD) has been demonstrated to induce ferroptosis in head and neck cancer cells by regulating glutamine metabolism [12]. Copper may inhibit the activity of glioblastoma by impacting the processes of apoptosis and DNA damage repair in glioblastoma cells [13]. Moreover, copper complexes exhibit anti-tumor cell proliferation effects by modifying the oxidative-reductive state of glioma [14]. Thus, targeting cuprotosis may offer a promising new approach to cancer therapy.

With the rapid development of gene sequencing technologies, bioinformatics analysis has become a promising option in cancer research. Since the role of cuprotosis in LGG remains unclear, we aimed to comprehensively explore the prognostic significance of cuprotosis in LGG. Based on the expression of cuprotosis genes, we employed consensus clustering to classify LGG samples into cuprotosis clusters A and B. The differences in prognosis, immune infiltration, and potential biofunction between cuprotosis clusters were explored using Kaplan–Meier survival analysis, ssGSEA, and GSVA. Next, we constructed a cuprotosis-related prognostic signature by analyzing the DEGs and survival data between cuprotosis cluster A and cluster B and confirmed its predictive accuracy for prognosis in LGG patients by internal validation and external validation. The relationship between the signature and the tumor microenvironment (TME), TMB, and immunotherapy response was also investigated.

## Methods

### Data acquisition

**Fig. 1:**
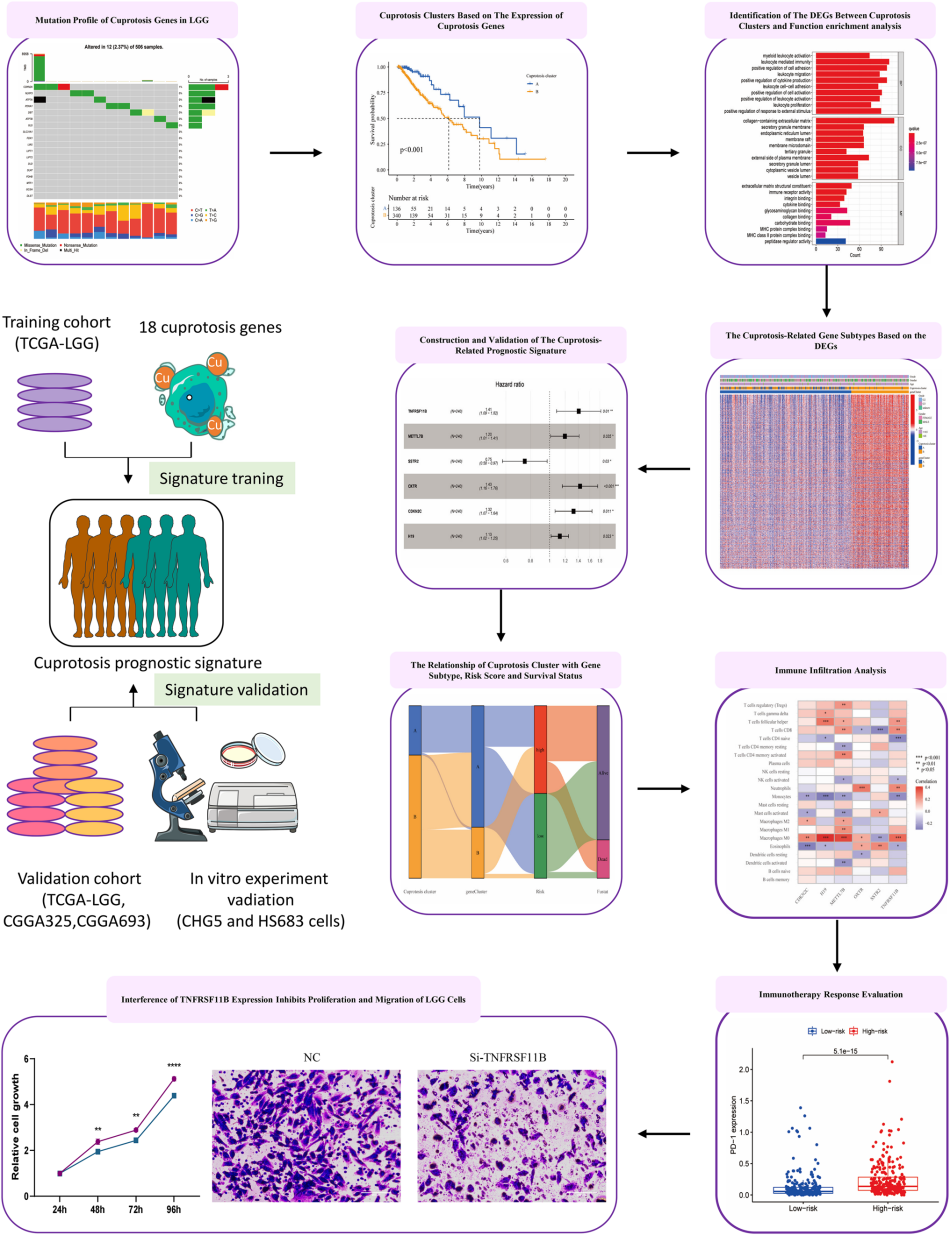
The flowchart of analyzing the cuprotosis genes in LGG

In this study, gene expression data, clinical information, and mutation data for LGG patients were obtained from The Cancer Genome Atlas (TCGA, https://portal.gdc.cancer.gov/) and the Chinese Glioma Genome Atlas (CGGA, http://www.cgga.org.cn/) databases [15, 16]. The transcriptome expression profile of 515 LGG cases was obtained from TCGA, along with clinical information including sex, age, OS, survival status, stage, and mutation data (Table 1). RNA sequencing data in FPKM format were converted to TPM format. Additionally, external validation data for LGG patients (186  LGG cases in mRNAseq_325 and 444 LGG cases in mRNAseq_693, Table 1) were obtained from CGGA, including RNA sequencing data and clinical information including sex, age, OS, survival status, stage, primary/recurrent, IDH mutation status, 1p19q codeletion status, radiotherapy, chemotherapy, and histology. Cuprotosis-related genes (NLRP3, ATP7A, ATP7B, SLC31A1, FDX1, LIAS, LIPT1, LIPT2, DLD, DLAT, PDHA1, PDHB, GLS, GCSH, MTF1, CDKN2A, DBT, and DLST) were derived from published literatures [8, 17–19]. All data were preprocessed by the “limma” and “sva” R packages [20]. The flowchart of the data analysis was shown in Fig. 1.

**Table 1:** The clinical information of LGG patients in TCGA and CGGA cohorts

	TCGA	CGGA	
	LGG-mRNA-seq	mRNA-seq_325	mRNA-seq_693
Total	515	186	444
Age			
≤ 65 years	483	183	440
> 65 years	32	3	3
Not reported	0	0	1
Gender			
Female	230	71	193
Male	285	115	251
Not reported	0	0	0
Pathological stage of Glioma			
WHO Stage II	249	103	188
WHO Stage III	265	79	255
Not reported	1	4	1
Survival status			
Alive	406	87	247
Dead	109	93	157
Not reported	0	6	40
P/R			
Primary	NA	144	282
Recurrent	NA	38	162
Not reported	NA	4	NA
IDH mutation status			
Wildtype	NA	51	96
Mutant	NA	134	307
Not reported	NA	1	41
1p19q codeletion status			
Codel	NA	60	132
Non-codel	NA	121	273
Not reported	NA	5	39
Radiotherapy			
Yes	NA	152	316
No	NA	25	87
Not reported	NA	9	41
Chemotherapy			
Yes	NA	85	266
No	NA	84	125
Not reported	NA	17	53
Histology			
A	NA	33	38
AA	NA	14	34
AO	NA	9	28
AOA	NA	27	82
O	NA	26	23
OA	NA	35	77
rA	NA	6	26
rAA	NA	14	31
rAO	NA	3	23
rAOA	NA	12	57
rOA	NA	3	17
rO	NA	0	7
NA	NA	4	1

### Mutation, copy number, and differential analysis of cuprotosis-related genes

We utilized the “maftools” R package to generate a waterfall plot that allowed us to examine the mutation frequency and mutation type of cuprotosis genes in each sample. In addition to this, we acquired copy number matrix files from the Xena website (https://xena.ucsc.edu/) to create a copy number circle diagram using the “CNVfreq” and “Rcirocs” R packages. The diagram helped us highlight the increase or deletion frequency of cuprotosis gene copy number variants (CNV). We then used the “Reshape2” and “ggpubr” R packages to identify cuprotosis genes with significant differences between normal and tumor tissues. Wilcoxon test was performed to compare the gene expression level, and *p* < 0.05 was identified as statistically significant.

### Consensus clustering analysis of cuprotosis genes

To cluster the LGG samples from TCGA and CGGA, we used the “Consensus Cluster Plus” package based on the expression of cuprotosis genes. The samples were divided into cuprotosis cluster A and cuprotosis cluster B, with k-values from 1 to 9. We selected the k-values with stable clustering ability based on the clustering effect, including a low variation coefficient, high consistency of clusters, and a relatively flat cumulative distribution function (CDF) curve [21, 22].

### Kaplan–Meier (KM) survival analysis and principal components analysis (PCA)

Kaplan–Meier survival analysis was performed to compare survival differences between the two cuprotosis clusters by the “Survival” and “Survminer” R packages. Additionally, we utilized the “ggplot2” R package to perform PCA analysis, which allowed us to observe the distribution of samples in the two cuprotosis clusters.

### Heatmap of cuprotosis clusters

To make clear the difference in the clinical characteristics between two cuprotosis clusters, we extracted the clinical information including tumor grade, sex, and age from TCGA and CGGA cohort and plotted a heatmap to visualize the distribution of cuprotosis genes and clinical characteristics across different cuprotosis clusters using the "pheatmap" R package.

### Gene set variation analysis and single-sample gene set enrichment analysis (GSVA and ssGSEA)

GSVA is a powerful tool for analyzing gene sets and identifying enrichments in biological pathways. It is an unsupervised and nonparametric method that scores gene sets and transforms them into the pathway level [23]. To carry out enrichment analysis in our study, we downloaded the "c2.cp.kegg.v7.2.symbols.gmt" gene set from MSigDB, a comprehensive collection of annotated gene sets. We then used the GSVA algorithm to calculate each gene set score and explore the biological function differences between the cuprotosis clusters that we were investigating. To ensure the statistical significance of our results, we set an adjusted *p* value of less than 0.05 as the threshold for determining significant enrichments. The ssGSEA algorithm is conducted on the basis of immune gene sets, including genes associated with various immune cell types, pathways, functions, and checkpoints. In this study, we used the ssGSEA algorithm via the “GSVA” R package to comprehensively evaluate the immunologic features of each LGG sample across different cuprotosis clusters [24].

### Identifying and clustering of DEGs between the cuprotosis clusters

DEGs between LGG patients in different cuprotosis clusters were screened using the “Limma” R package, and the significance criteria were set as | logFC |> 0.585 and FDR < 0.05 [20]. A Venn diagram of DEGs was plotted using the “VennDiagram” R package. Univariate Cox regression analysis was used to identify prognostic cuprotosis DEGs with *p* < 0.05 [25]. Then, in light of the prognostic cuprotosis DEGs, consistent clustering analysis was used to categorize LGG patients into distinct gene subtypes via the “Consensus Cluster Plus” R package [21]. Finally, KM survival analysis was performed to compare survival differences between the gene subtypes.

### Functional enrichment analysis

The "clusterProfiler" and “enrichplot” R packages were used to conduct Kyoto Encyclopedia of Genes and Genomes (KEGG) pathway and Gene Ontology (GO) enrichment analyses [26, 27]. The KEGG pathway enrichment analysis identified the signal transduction pathways and immune-related pathways in which cuprotosis clustering DEGs were significantly enriched. GO functional analysis includes biological processes, cellular component, and molecular function analysis.

### Heatmap and cuprotosis gene expression of gene subtypes

We plotted a heatmap to visually represent the distribution of clinical characteristics such as sex, age, grade, and cuprotosis clusters in different gene subtypes using the “pheatmap” R package. “Reshape2”, “ggpubr”, and “ggplot2” R packages were used to compare the cuprotosis gene expression differences between gene subtypes.

### Cuprotosis-related prognostic signature construction

According to the results of the univariate Cox analysis, we identified 1278 prognosis-related DEGs (*p* < 0.05). Then, we randomly divided the LGG samples from TCGA database into the training and internal validation groups. After the removal of highly correlated genes from the 1278 prognosis-related DEGs through the LASSO algorithm using the “glmnet” R package, a multivariate Cox regression analysis was then applied to the remaining genes to establish a prognostic signature in the training group. For each patient, the risk score was calculated based on the following formula:

$$Cuprotosis\;risk\;score\; = \sum {(Expi\; \times \;Coefi)}$$

(Expi denotes each signature gene’s expression level, and Coefi denotes the corresponding coefficient.)

The reliability of the prognostic signature was validated using the internal validation (TCGA-LGG) and external validation groups (CGGA325 and CGGA693). All samples were divided into high-risk and low-risk groups according to the median risk score of the training group. In both training and validation groups, the signature’s predictive capability was assessed by KM survival analysis and ROC curves using the “timeROC”, “survival”, and "survminer" R packages. Additionally, we also created a nomogram using the "survival", "rms", and "regplot" R packages to predict the 1-, 3-, and 5-year survival rates and calibrated the signature to evaluate its consistency with practice.

### Sankey diagram

To visualize the correspondence of the cuprotosis cluster with geneCluster, risk score, and patient survival outcome, we used the “ggplot2”, “ggalluvial” and “dplyr” R packages to plot the Sankey diagram. Meanwhile, the “ggpubr” R package was applied to further analyze and compare the risk scores of cuprotosis clusters and gene subtypes. For further analysis, we loaded the "reshape2", "ggpubr", and "ggplot2" packages to detect the expression differences of cuprotosis genes between high- and low-risk groups.

### Correlation of the prognostic signature with TME, genetic mutation, and immune checkpoints

“Reshape2”, “tidyverse”, “ggplot2”, “ggpubr” and “ggExtra” R packages were used to analyze the correlation of risk score with immune cells and plot a heatmap depicting the relationship of signature genes with immune cells. Additionally, we compared the TME scores between high- and low-risk groups using the “reshape2” and “ggpubr” R packages. To further investigate the signature’s predictive capability in immunotherapy response, we analyzed the expression of immune checkpoints such as PD-1, PD-L1, CTLA4, LAG3, TIM-3, and GAL9 between high- and low-risk groups using “limma”, “ggplot2”, “ggpubr” and “ggExtra” R packages [28–30].

### siRNA treatment

Normal human astrocyte line HA1800 and LGG cell lines CHG5 and HS683 were purchased from the American Type Culture Collection (Manassas, VA, USA). TNFRSF11B siRNA (GeneCodex, Wuhan, China) was transfected into CHG5 and HS683 cells with InvitroRNA™ (InvivoGene Biotechnology, Suzhou, China).

### qRT-PCR

To detect the gene expression of TNFRSF11B and the changes resulting from TNFRSF11B siRNA transfection, we performed qRT-PCR analysis. We extracted cellular RNA from HA1800, CHG5, HS683, CHG5 transfected siRNA, and HS683 transfected siRNA using TRIzol reagent (TaKaRa, Japan). Then, we employed the HiScript II qRT SuperMix (Vazyme, China) to synthesize cDNA and conduct qRT-RCR using the ChamQ Universal SYBR qPCR Master Mix (Vazyme, China). The primer sequences used in the qRT-PCR analysis were: TNFRSF11B-Forward: CACAAATTGCAGTGTCTTTGGTC; TNFRSF11B-Reverse: TCTGCGTTTACTTTGGTGCCA; β-actin-Forward: TCCTCTCCCAAGTCCACACAGG; GAPDH-Reverse: GGGCACGAAGGCTCATCATTC.

### CCK8 assay

We seeded CHG5 and HS683 cells into 96-well plates after transfecting them with TNFRSF11B siRNA for 24 h and then treated them with CCK8 reagent (MCE, USA) according to the manufacturer's instruction. After 24, 48, 72, and 96 h, the OD450 values of CHG5 and HS683 cells were detected via a microplate reader (BioTek, USA).

### Wound healing assay

After being transfected with TNFRSF11B siRNA for 24 h, CHG5 and HS683 cells were seeded into a 6-well plate and then scraped with a 1 ml pipette tip. Cell migration images were captured at 0, 24, 36, and 48 h after scratching.

### Transwell migration assay

After being transfected with TNFRSF11B siRNA for 24 h, CHG5 and HS683 cells were cultured in the upper chambers with 200 µl medium without serum, and the lower chambers were filled with 500 µl medium containing 20% fetal bovine serum. After 24 h of incubation at 37°, the cells in the lower chamber were fixed with 4% paraformaldehyde, stained with 0.1% crystal violet, and imaged using light microscopy. The number of migratory cells was counted and recorded.

### Statistical analyses

For the data comparison between the two groups, we adopted the t-test for variables with a normal distribution and the Wilcoxon rank sum test for variables conforming to non-normal distribution. For the data comparison among more than two groups, we employed the one-way ANOVA test as a parametric method and the Kruskal–Wallis test as a non-parametric method. To determine the cutoff score of the risk score, we applied the surv-cutpoint function. The survival analysis was performed through the Kaplan–Meier method. Additionally, we developed the prognostic signature using the univariate Cox-LASSO-multivariate Cox regression analysis method [31]. *p* < 0.05 was identified as statistical significance. We performed all statistical analyses using the R software (version 4.2.0) and GraphPad Prism software (version 7.0).

## Results

### Mutation profile of cuprotosis genes in LGG

**Fig. 2:**
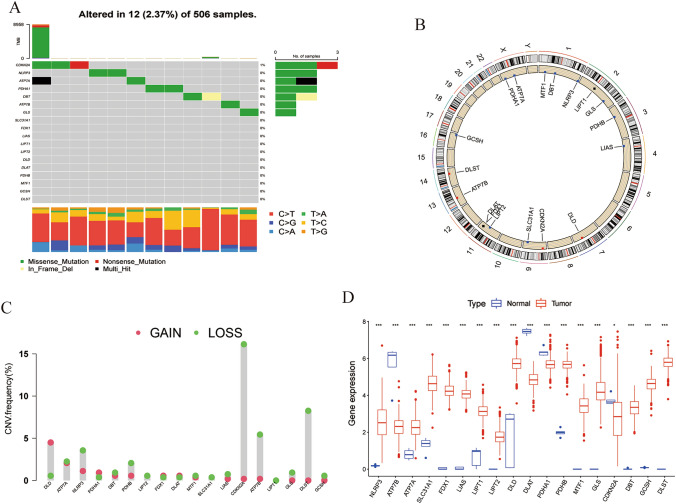
Landscape of gene mutation and CNV of 18 cuprotosis genes in LGG. **A** Gene mutation waterfall diagram of 18 cuprotosis genes in LGG patients. **B** Location of CNV alterations of the cuprotosis genes on chromosomes in the TCGA-LGG cohort. **C** Frequency of CNV alterations in cuprotosis genes. Red dots represented CNV amplification, while green dots represented CNV deletion. **D** Differential expression of 18 cuprotosis genes between normal tissue and LGG tissues. (*, *p* < 0.05; ***, *p* < 0.001) (Color figure online)

In this study, we first focused on analyzing the gene mutations of 18 cuprotosis genes (NLRP3, ATP7A, ATP7B, SLC31A1, FDX1, LIAS, LIPT1, LIPT2, DLD, DLAT, PDHA1, PDHB, GLS, GCSH, MTF1, CDKN2A, DBT, and DLST) in patients with LGG, and observed a low frequency of mutations in LGG samples. Out of 506 samples, only 12 (2.37%) had alterations in cuprotosis genes, and these alterations had low frequencies of ≤ 1% (Fig. 2A). Specifically, CDKN2A had the highest mutation frequency of 1%, while the remaining genes showed no mutation (Fig. 2A). The CNV alterations' location on the chromosome for cuprotosis genes was also analyzed (Fig. 2B), and CNV deletions commonly occurred in CDKN2A, DLST, and ATP7B genes, whereas CNV amplification occurred more frequently in DLD and PDHA1 genes (Fig. 2C). Additionally, we found that the expression of 14 of the 18 genes was significantly upregulated in LGG tissues compared to normal tissues, while ATP7B, DLAT, PDHA1, and CDKN2A were significantly downregulated (Fig. 2D).

### Immune cell infiltration analysis between cuprotosis clusters

**Fig. 3:**
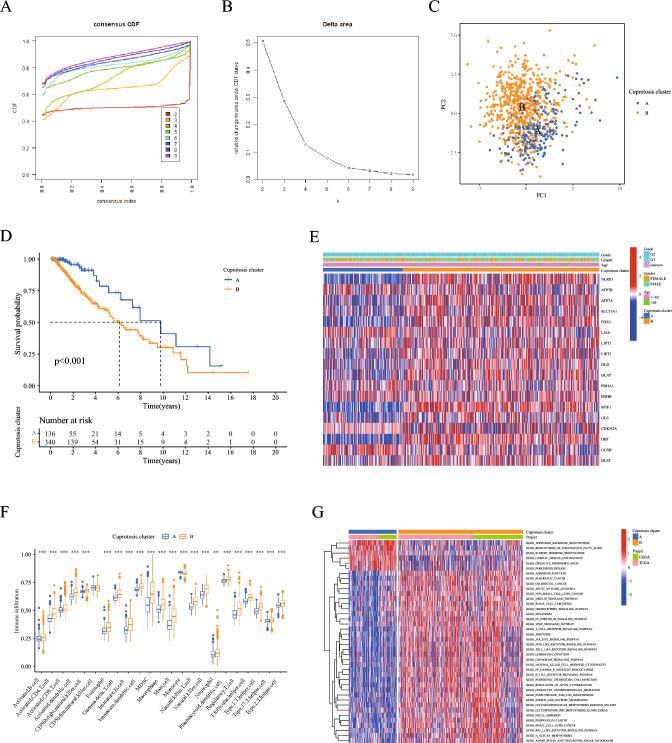
Immune cell infiltration and functional analysis between cuprotosis clusters. **A** Cumulative distribution function (CDF) curves displayed consensus distributions from k = 2 to k = 9. **B** Delta area curves represented the number of classes k in each category versus relative changes in the area under the CDF curves for k-1. The horizontal axis indicated the number of categories (k), while the vertical axis indicated the relative changes in the area under the CDF curves. **C** PCA analysis showed the distribution of samples in cuprotosis cluster A and cuprotosis cluster B. **D** Kaplan–Meier survival curves between cuprotosis clusters. **E** A heatmap showed the distribution of 18 cuprotosis genes among different grades, sexes, ages, and cuprotosis clusters. **F** Differences in the infiltration of 23 immune cells between cuprotosis clusters. **G** GSVA pathway enrichment analysis between cuprotosis clusters, red represented activated pathways, while blue represented inhibited pathways in the heatmap

To delve deeper into the understanding of the biological processes and clinical significance of cuprotosis genes, we conducted a consensus clustering analysis on samples from TCGA and CGGA databases. The categorization of these samples was based on the expression levels of 18 cuprotosis genes. We tested different k values ranging from 2 to 9 and found that k = 2 provided the best classification stability (Fig. 3A, B). As such, we identified two distinct groups: cuprotosis cluster A and cuprotosis cluster B. PCA revealed that most LGG patients can be differentiated based on cuprotosis clustering (Fig. 3C). The KM survival curve provided further insight, showing a significant difference in OS between the two cuprotosis clusters. Patients in cuprotosis cluster A had a more favorable prognosis than those in cuprotosis cluster B (Fig. 3D). The heatmap visually represented the distribution of the 18 cuprotosis genes across samples in different clusters, grades, ages, and genders. Importantly, the results indicated that the percentage of grade 3 LGG patients and most of cuprotosis genes’ expression were relatively higher in cuprotisis cluster B (Fig. 3E).

In addition, we also identified significant differences in immune cell infiltration between the cuprotosis clusters. Compared to cuprotosis cluster A, patients in cuprotosis cluster B had a higher level of immune cell infiltration, such as activated B cells, activated CD4 T cells, activated CD8 T cells,activated dendritic cell, CD56 bright natural killer (NK) cell, etc. On the other hand, monocytes were more abundant in cuprotosis cluster A (Fig. 3F). To gain more insights into the biological functions that distinguish the two cuprotosis clusters, a GSVA enrichment analysis was performed. The analysis showed that immune-related pathways, including the B cell receptor signaling pathway, T cell receptor signaling pathway, chemokine signaling pathway, and tumor-related pathways such as the JAK-STAT signaling pathway, were significantly enriched in cuprotosis cluster B (Fig. 3G). Conversely, pathways such as terpenoid backbone biosynthesis, biosynthesis of unsaturated fatty acids, and oxidative phosphorylation were mainly enriched in cuprotosis cluster A (Fig. 3G), indicating that these pathways may play a pivotal role in mediating the clinical outcome differences between the two clusters.

### Identification of cuprotosis-related DEG subtypes in LGG

**Fig. 4:**
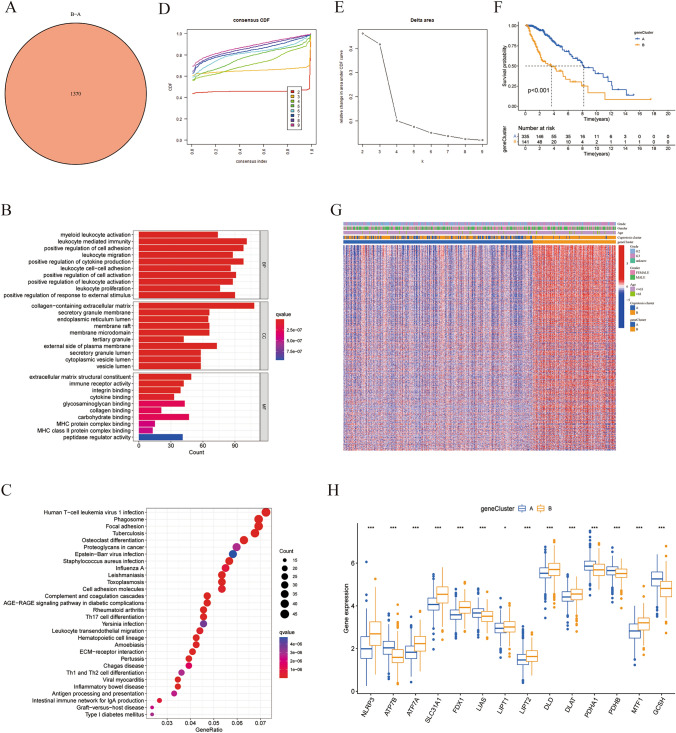
Identification of cuprotosis-related DEGs subtypes in LGG. **A** Venn diagram of differential genes between cuprotosis cluster A and cuprotosis cluster B. **B** Bar graph of GO functional enrichment analysis for the DEGs between cuprotosis clusters. **C** Bubble plot of KEGG pathway enrichment analysis for the DEGs between cuprotosis clusters. **D** CDF curves displayed consensus distributions from k = 2 to k = 9. **E** Delta area curves represented the number of classes k in each category versus relative changes in the area under the CDF curves for k-1. The horizontal axis indicated the number of categories (k), while the vertical axis indicated the relative changes in the area under the CDF curves. **F** Kaplan–Meier survival curves between different gene subtypes. **G** A heatmap showed the distribution of DEGs among LGG patients of different grades, sexes, ages, cuprotosis clusters, and gene subtypes. **H** Differential expression of cuprotosis genes between geneCluster A and B

After observing the significant survival differences between the two cuprotosis clusters, we were curious about whether genetic differences played a crucial role. To investigate this possibility, we carried out an extensive analysis to identify potential genetic alterations between the two cuprotosis clusters. The analysis revealed that there were 1370 differentially expressed genes (DEGs) between cuprotosis cluster A and B (Fig. 4A). In terms of GO functional enrichment analysis, the DEGs were mainly enriched in leukocyte-mediated immunity, positive regulation of cell adhesion and cytokine production for biological process (BP), collagen-containing extracellular matrix for cellular components (CC), extracellular matrix structural constituent, and carbohydrate-binding for molecular function (MF) (Fig. 4B). We also performed KEGG pathway enrichment analysis and found that the DEGs were mainly enriched in pathways related to human T-cell leukemia virus 1 infection, phagosome, focal adhesion, tuberculosis, osteoclast differentiation, and proteoglycans in cancer (Fig. 4C). These findings suggest that there are significant genetic differences between the two cuprotosis clusters and that these differences may be contributing to the observed survival disparities.

To further explore the role of specific DEGs in the clinical characteristics and cuprotosis clusters, we performed consensus clustering analysis based on the DEGs. From k = 2 to k = 9, we found that k = 2 provided the best clustering stability (Fig. 4D, E). Therefore, we identified two cuprotosis-associated gene subtypes, namely, geneCluster A and geneCluster B. KM survival analysis revealed a significant survival advantage for geneCluster A over geneCluster B (Fig. 4F). Furthermore, the heatmap revealed that LGG patients in the cuprotosis clusters and gene subtypes exhibited significant variations in tumor grade, gender, and age. Intriguingly, a higher proportion of patients with grade 3 or above 65 years old were found in cuprotosis cluster B and geneCluster B (Fig. 4G).Additionally, the expression differences of cuprotosis genes in different gene subtypes were analyzed. We identified 9 cuprotosis genes, namely, NLRP3, ATP7A, SLC31A1, FDX1, LIPT1, LIPT2, DLD, DLAT, and MTF1, were significantly distributed in geneCluster B. On the other hand, 5 cuprotosis genes, namely ATP7B, LIAS, PDHA1, PDHB, and GCSH showed an upregulated situation in geneCluster A (Fig. 4H). Overall, these findings demonstrate the importance of DEGs in cuprotosis clustering and their relationship with different clinical characteristics.

### Construction and validation of the cuprotosis-related prognostic signature

**Fig. 5:**
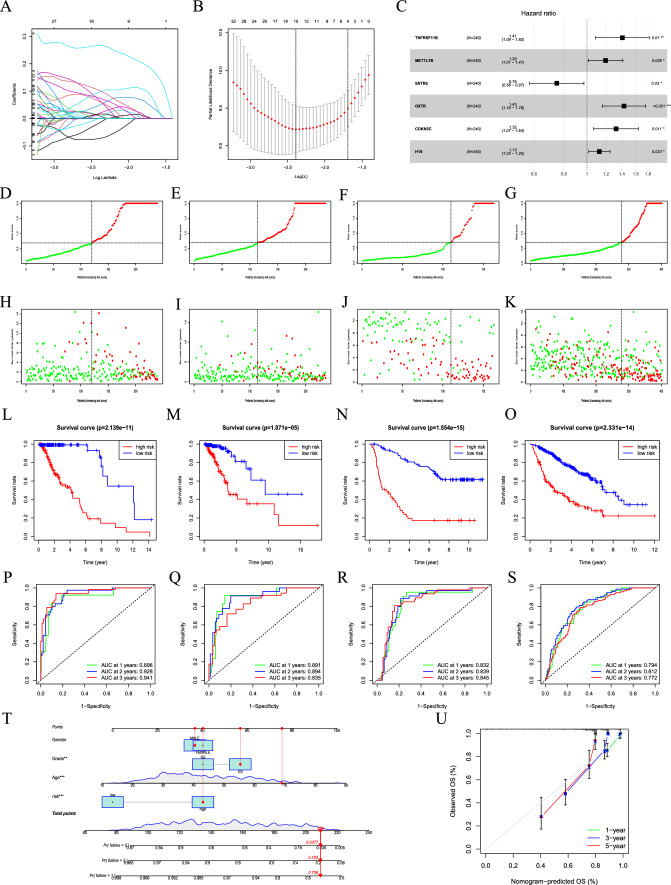
Construction of a prognostic signature to predict LGG patients' prognosis based on the DEGs between cuprptosis clusters. **A** Lasso coefficient plot. **B** The best log Lambda value was selected in the training group via tenfold cross-validation. **C** The forest map visually showed the HR value and 95% confidence interval for all signature genes. **D** LGG patients in the training group were classified into high-risk and low-risk groups based on the median cut-off risk score of the training group. **E** LGG patients in the internal validation group were classified into high-risk and low-risk groups based on the median risk score of the training group. **F** LGG patients in the external validation group (CGGA325) were classified into high-risk and low-risk groups based on the median risk score of the training group. **G** LGG patients in the external validation group (CGGA693) were classified into high-risk and low-risk groups based on the median risk score of the training group. **H** Survival status distribution of LGG patients with different risks in the training group. **I** Survival status distribution of LGG patients with different risks in the internal validation group. **J** Survival status distribution of LGG patients with different risks in the external validation group (CGGA325). **K **Survival status distribution of LGG patients with different risks in the external validation group (CGGA693). **L** Kaplan–Meier survival curves between high-risk and low-risk groups in the training group. **M** Kaplan–Meier survival curves between high-risk and low-risk groups in the internal validation group. **N** Kaplan–Meier survival curves between high-risk and low-risk groups in the external validation group (CGGA325). **O** Kaplan–Meier survival curves between high-risk and low-risk groups in the external validation group (CGGA693). **P** Time-ROC curves of the training group. **Q** Time-ROC curves of the internal validation group. **R** Time-ROC curves of the external validation group (CGGA325). **S** Time-ROC curves of the external validation group (CGGA693). **T** A nomogram to predict the 1-, 3-, and 5-year OS. **U** Calibration curves to determine the predictive accuracy of the nomogram

Considering the complexity and heterogeneity of each LGG patient, we constructed a cuprotosis-related prognostic signature to judge the prognosis of LGG patients. The TCGA-LGG samples were randomly divided into the training and internal validation groups, while the CGGA325 and CGGA693 samples were used as external validation groups. For constructing the prognostic signature, we first identified 1278 prognosis-related DEGs between the two cuprotosis clusters through univariate Cox analysis. The LASSO analysis was then conducted on these genes for in-depth shrinkage and selection. After removing the highly correlated genes by the lasso algorithm, a multivariate Cox regression analysis was then used to construct the signature (Fig. 5A, B). A total of six DEGs were identified, and their corresponding coefficients were obtained (Fig. 5C, Table 2). For each LGG patient, the risk score was calculated based on the following formula: Risk score = 0.3413* TNFRSF11B + 0.1794* METTL7B-0.2905* SSTR2 + 0.3566* OXTR + 0.2803* CDKN2C + 0.1194* H19. Patients in the training, internal validation cohort, and two external validation cohorts (CGGA325 and CGGA693) were categorized into high- and low-risk groups based on the median score of the training group, respectively (Fig. 5D-G). The survival status analysis showed that high-risk patients were more liable to have a less favorable prognosis than patients in the low-risk group in all four cohorts (Fig. 5H-K). Additionally, KM survival analysis indicated that LGG patients at high risk had a poorer OS than those at low risk in all four cohorts (*p* = 2.139e-11, *p* = 1.071e-5, *p* = 1.554e-15, *p* = 2.331e-14, Fig. 5L-O). We then tested our cuprotosis signature’s prognostic ability through ROC analysis. The AUC in the training group was 0.896 at 1 year, 0.928 at 2 years, and 0.941 at 3 years (Fig. 5P), while in the internal validation group was 0.891 at 1 year, 0.894 at 2 years, and 0.835 at 3 years (Fig. 5Q). Additionally, the signature expressed stable prognostic capability in the external validation groups as well. In the CGGA325 cohort, the AUC was 0.832 at 1 year, 0.839 at 2 years, and 0.845 at 3 years (Fig. 5R), while in the CGGA693 cohort was 0.794, 0.812, and 0.772 at 1, 2, and 3 years, respectively (Fig. 5S). Generally, the AUC in the four cohorts reached more than 0.75, and most of them were over 0.8, hinting that our signature is a reliable predictor of prognosis in LGG patients. Furthermore, a nomogram was plotted to predict the 1-, 3-, and 5-year OS according to each patient’s gender, grade, age, and risk score (Fig. 5T). Figure 5T showed that the patient’s total score of gender, grade, age, and risk score was 209, corresponding to survival rates of 73.8%, 18.3%, and 3.77% for 1, 3, and 5 years, respectively. The nomogram was found to accurately predict the OS based on the calibration curve. Overall, the findings suggest that the cuprotosis signature is a stable predictive factor for prognosis in LGG and that the nomogram can provide useful information for predicting the patient’s OS.

**Table 2:** Multivariate Cox regression analysis results of model genes

Gene	Coef	HR	HR.95L	HR.95H	*p* value
TNFRSF11B	0.341339	1.406831	1.085852	1.822691	0.009786
METTL7B	0.179374	1.196468	1.012499	1.413864	0.035222
SSTR2	− 0.29048	0.747901	0.575657	0.971684	0.029627
OXTR	0.356585	1.428444	1.160804	1.757791	0.000755
CDKN2C	0.280349	1.323592	1.065979	1.643462	0.011133
H19	0.119443	1.126869	1.016273	1.249502	0.023438

### The relationship of cuprotosis cluster with geneCluster, risk score, and survival status

**Fig. 6:**
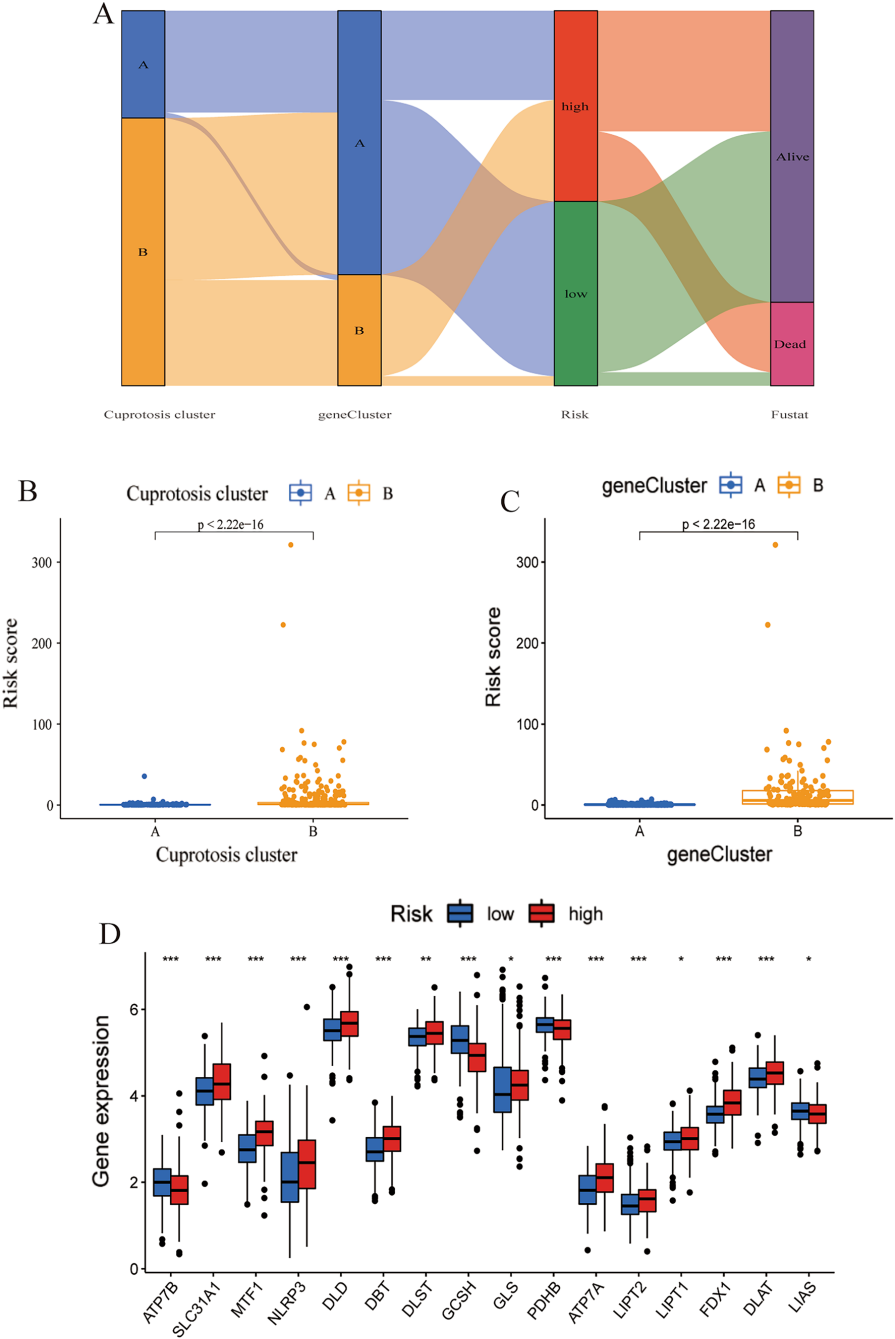
The relationship of cuprotosis cluster with gene subtype, risk score, and survival status. **A** A Sankey diagram showed the correspondence of cuprotosis cluster, gene subtype, risk score, and survival status. **B** Comparison of the risk scores between cuprotosis clusters. **C** Comparison of the risk scores between gene subtypes. **D** Differences in cuprotosis gene expression between high-risk and low-risk groups

A Sankey diagram was used to visually depict the distribution of LGG samples among different classification methods. The results showed that the majority of patients in cuprotosis cluster A was associated with geneCluster A, which had a lower risk score and a better prognosis (Fig. 6A). In contrast, most patients in geneCluster B corresponded to cuprotosis cluster B and had a higher risk score and poorer prognosis (Fig. 6A). This was supported by quantitative analysis, which showed a higher risk score in cuprotosis cluster B than cluster A (*p* < 2.22e-16, Fig. 6B). Additionally, geneCluster B also had a higher risk score (*p* < 2.22e-16, Fig. 6C), indicating a potential association between cuprotosis genes and prognosis in LGG. Further examination of this relationship revealed that 12 cuprotosis genes were significantly upregulated in high-risk groups, including SLC31A1 (*p* < 0.001), MTF1 (*p* < 0.001), NLRP3 (*p* < 0.001), DLD (*p* < 0.001), DBT (*p* < 0.001), DLST (*p* < 0.01), GLS (*p* < 0.05), ATP7A (*p* < 0.001), LIPT2 (*p* < 0.001), LIPT1 (*p* < 0.05), FDX1 (*p* < 0.001), and DLAT (*p* < 0.001), while ATP7B (*p* < 0.001), GCSH (*p* < 0.001), PDHB (*p* < 0.001), and LIAS (*p* < 0.05) were down-regulated in this group (Fig. 6D). These results suggest that these cuprotosis genes may contribute to the poor prognosis associated with high-risk LGGs.

### The cuprotosis-related prognostic signature characterized by distinct immune infiltration landscapes

**Fig. 7:**
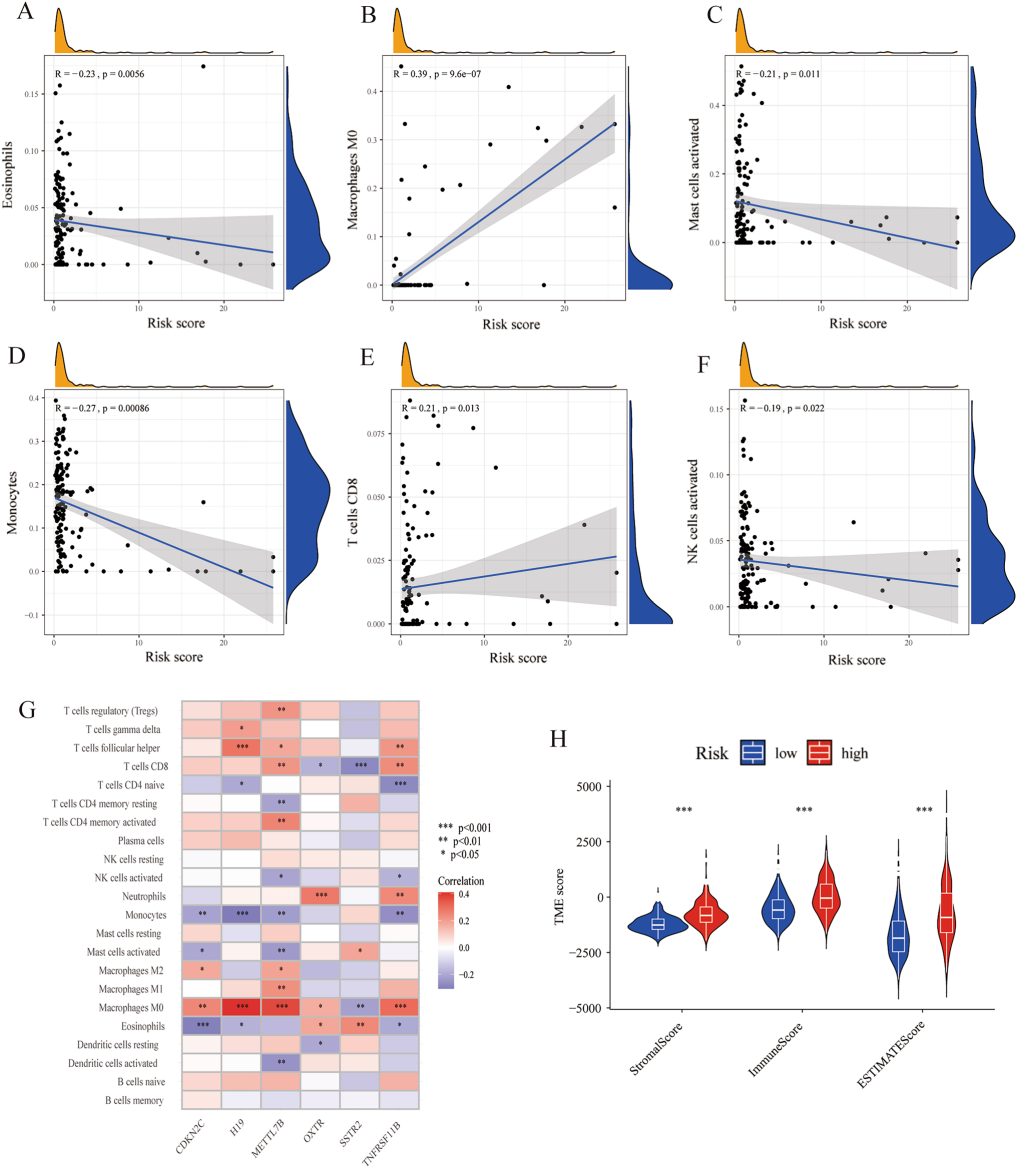
The cuprotosis-related prognostic signature was characterized by distinct immune infiltration landscapes. **A**–**G** Correlation of risk score with the infiltration of eosinophils, M0 macrophages, activated mast cells, monocytes, CD8 T cells, and activated NK cells. **H** Heatmap of correlation between the signature genes and 22 immune cell infiltration. Red represented the positive correlation, while blue represented the negative correlation. **I** Comparison of TME scores between high-risk and low-risk groups. (*, *p* < 0.05; **, *p* < 0.01; ***, *p* < 0.001) (Color figure online)

The above results revealed the differences in immune cell infiltration and immune-related pathways between cuprotosis clusters. To investigate whether cuprotosis affects LGG prognosis by influencing the TME, we analyzed the correlation of the risk score with immune cell infiltrations. The results showed that eosinophils (R = − 0.23, *p* = 0.0056, Fig. 7A), activated mast cells (R = − 0.21, *p* = 0.011, Fig. 7C), monocytes (R = − 0.27, *p* = 0.00086, Fig. 7D), and activated NK cells (R = − 0.19, *p* = 0.022, Fig. 7F) were negatively correlated with the risk score, whereas M0 macrophages (R = 0.39, *p* = 9.6e-7, Fig. 7B) and CD8 T cells (R = 0.21, *p* = 0.013, Fig. 7E) were positively correlated with the risk score. Furthermore, we plotted a heatmap to visually demonstrate the correlation between signature genes and the 22 immune cell infiltrations, among which M0 macrophage was the only cell type that was associated with all of the 6 signature genes: CDKN2C (*p* < 0.01, Fig. 7G), H19 (*p* < 0.001, Fig. 7G), METTL7B (*p* < 0.001, Fig. 7G), OXTR (*p* < 0.05, Fig. 7G), SSTR2 (*p* < 0.01, Fig. 7G), and TNFRSF11B (*p* < 0.001, Fig. 7G). In contrast, resting NK cells, plasma cells, naïve B cells, and memory B cells did not have any correlations with signature genes. We also found METTL7B to be the most significant gene associated with immune cell infiltration. We identified 12 types of immune cells that had significant correlations with METTL7B, including regulatory T cells (*p* < 0.01, Fig. 7G), T follicular helper cells (*p* < 0.05, Fig. 7G), CD8 T cells (*p* < 0.01, Fig. 7G), resting memory CD4 T cells (*p* < 0.01, Fig. 7G), activated memory CD4 T cells (*p* < 0.01, Fig. 7G), activated NK cells (*p* < 0.05, Fig. 7G), monocytes (*p* < 0.01, Fig. 7G), activated mast cells (*p* < 0.01, Fig. 7G), M2 macrophages (*p* < 0.05, Fig. 7G), M1 macrophages (*p* < 0.01, Fig. 7G), M0 macrophages (*p* < 0.001, Fig. 7G), and activated dendritic cells (*p* < 0.01, Fig. 7G). Moreover, we used the ESTIMATE algorithm to measure the stromal scores, immune scores, and ESTIMATE scores of LGG specimens and found the high-risk group had significantly higher stromal scores, immune scores, and ESTIMATE scores, indicating a lower level of tumor purity, and a higher number of immune and stromal cells in the high-risk group (Fig. 7H). These findings revealed that the cuprotosis signature might be related to the immunological status of LGG.

### Cuprotosis-related prognostic signature predicted the efficacy of immunotherapy

**Fig. 8:**
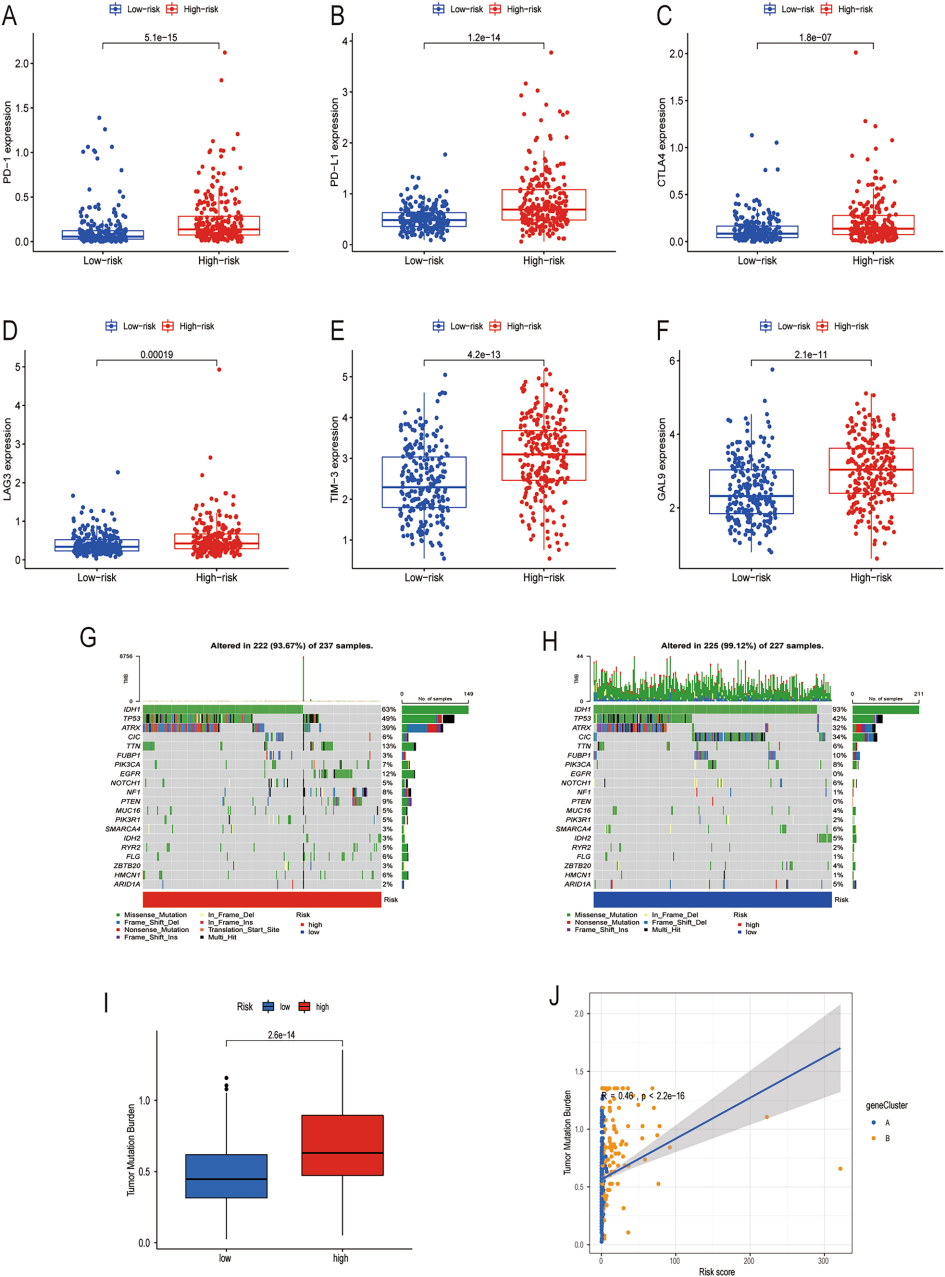
Cuprotosis-related prognostic signature predicted the efficacy of immunotherapy. **A**–**F **Differential expression of PD-1, PD-L1, CTLA4, LAG3, TIM-3, and GAL9 between high-risk and low-risk groups. **G** Mutation waterfall plot in the high-risk group. **H** Mutation waterfall plot in the low-risk group. **I** Comparison of TMB between high-risk and low-risk groups. **J** Correlation analysis of TMB and risk score

The use of immune checkpoint inhibitors (ICIs) targeting PD-1, PD-L1, and CTLA-4 has become a promising treatment strategy for various types of cancer. To further explore the correlation between the cuprotosis signature and immunotherapy, the expression levels of various immune checkpoints were examined in high-risk and low-risk groups. Results indicated that patients in the high-risk group had significantly higher expression levels of PD-1 (*p* = 5.1e-15, Fig. 8A), PD-L1 (*p* = 1.2e-14, Fig. 8B), CTLA4 (*p* = 1.8e-07, Fig. 8C), LAG3 (*p* = 0.00019, Fig. 8D), TIM-3 (*p* = 4.2e-13, Fig. 8E), and GAL9 (*p* = 2.1e-11, Fig. 8F) compared to those in the low-risk group [32–35]. Additionally, we also revealed a positive correlation between risk score and the expressions of PD-1 (R = 0.44, *p* < 2.2e-16, Supplementary Fig. 1A), PD-L1 (R = 0.48, *p* < 2.2e-16, Supplementary Fig. 1B), CTLA4 (R = 0.3, *p* = 1.4e-11, Supplementary Fig. 1C), LAG3 (R = 0.16, *p* = 0.00046, Supplementary Fig. 1D), TIM-3 (R = 0.41, *p* < 2.2e-16, Supplementary Fig. 1E), and GAL9 (R = 0.36, *p* = 4.3e-16, Supplementary Fig. 1F). According to previous studies, it is widely recognized that tumors with high TMB tend to respond positively to immunotherapy and have a more favorable prognosis [36, 37]. Based on the mutation data of the TCGA-LGG cohort, we plotted the waterfall diagrams to visually represent the distribution of somatic mutations in both high-risk and low-risk groups. In the high-risk group, 222 (93.67%) of 237 samples experienced somatic mutations (Fig. 8G), while 225 (99.12%) of 227 samples mutated in the low-risk group (Fig. 8H). According to the data presented in Fig. 8G, the ten genes with the highest frequency of mutations in the high-risk group were IDH1, TP53, ATRX, TTN, EGFR, PTEN, NF1, PIK3CA, CIC, and FLG. IDH1 had the highest frequency of mutations at 63%, followed by TP53 at 49% and ATRX at 39%. The remaining genes had lower mutational frequencies, with TTN at 13%, EGFR at 12%, PTEN at 9%, NF1 at 8%, PIK3CA at 7%, CIC at 6%, and FLG at 6%. On the other hand, the low-risk group had a different set of highly mutated genes, according to Fig. 8H. IDH1 remained the most frequently mutated gene at 93%, followed by TP53 at 42% and CIC at 34%. ATRX was also highly mutated in this group at 32%. The remaining genes with high mutational frequencies were FUBP1 at 10%, PIK3CA at 8%, TTN at 6%, NOTCH1 at 6%, SMARCA4 at 6%, and IDH2 at 5%. Therefore, IDH1, TP53, and ATRX were the genes with the highest mutation frequency in both groups. Specifically, IDH1 was the most frequently mutated gene, with it being mutated in 63% of samples in the high-risk group, and in 93% of samples in the low-risk group. A previous study showed that IDH1 wild-type glioma was prone to have a worse prognosis [38]. The differential analysis showed that the high-risk group had a significantly higher TMB (*p* = 2.6e-14, Fig. 8I). Furthermore, the correlation analysis suggested that there was a positive correlation between risk score and TMB (R = 0.46, *p* < 2.2e-16, Fig. 8J). Overall, these findings suggest that patients in the high-risk group may have an immunotherapeutic advantage over those in the low-risk group, given their higher TMB and expression of immune checkpoint molecules.

### Interference with TNFRSF11B expression inhibits proliferation and migration of LGG cells

**Fig. 9:**
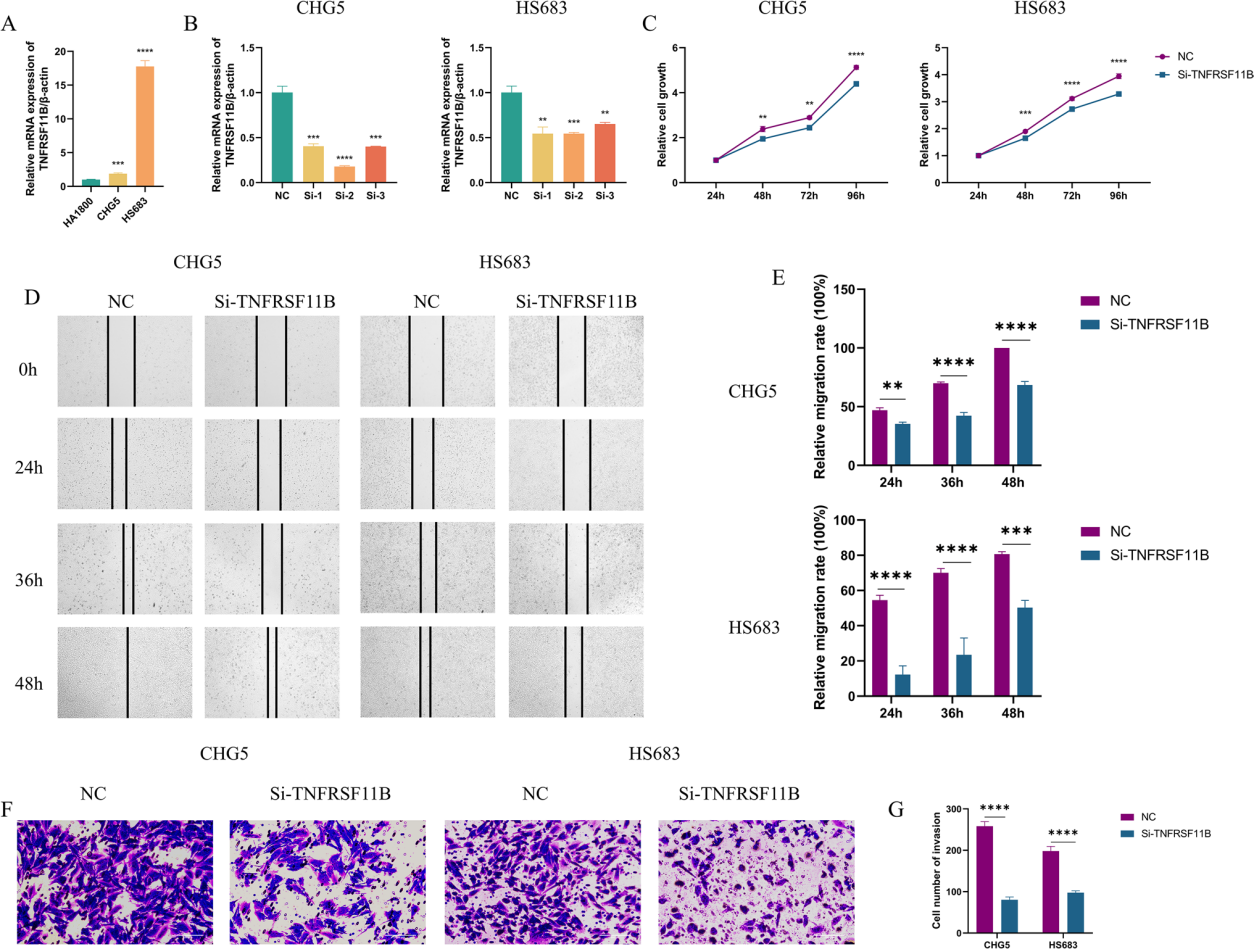
Interference of TNFRSF11B inhibits the proliferation and migration of LGG cells. **A** The expression of TNFRSF11B in the normal human astrocyte line HA1800 and LGG cell lines CHG5 and HS683 was detected by qRT-PCR. **B** The transfection efficiency of TNFRSF11B siRNA in CHG5 and HS683 cells was detected by qRT-PCR. **C** The CCK8 assay showed that TNFRSF11B interference significantly inhibited CHG5 and HS683 cell proliferation at 48 h, 72 h, and 96 h. **D** Representative images of CHG5 and HS683 cells at 0 h, 24 h, 36 h, and 48 h in the wound healing assay. **E** The result of the wound healing assay showed that interference with TNFRSF11B significantly inhibited the migration ability of CHG5 and HS683 cells. **F** Representative images of CHG5 and HS683 cells in the transwell migration assay. **G** The result of the transwell migration assay showed that interference with TNFRSF11B significantly inhibited the migration ability of CHG5 and HS683 cells. *p < 0.05, **p < 0.01, ***p < 0.001, ****p < 0.0001

To further increase the credibility of the prognostic signature, we conducted functional validation through in vitro experiments. Since the previous studies have explored the role of the signature genes METTL7B, SSTR2, OXTR, CDKN2C, and H19 in glioma, while TNFRSF11B has been poorly studied, we examined the functional impact of TNFRSF11B on LGG cell behavior [39–44]. Our investigation revealed that TNFRSF11B mRNA expression levels were higher in CHG5 (*p* < 0.001, Fig. 9A) and HS683 (*p* < 0.0001, Fig. 9A) cells compared to normal human astrocyte line HA1800 cells. To interfere with TNFRSF11B expression, we used three different siRNAs and were able to successfully reduce TNFRSF11B expression levels in both CHG5 and HS683 cells (Fig. 9B). Using the CCK8 assay, we found that TNFRSF11B interference significantly inhibited CHG5 and HS683 cell proliferation at 48 h (*p* < 0.01,* p* < 0.001, Fig. 9C), 72 h (*p* < 0.01,* p* < 0.0001, Fig. 9C) and 96 h (*p* < 0.0001,* p* < 0.0001, Fig. 9C). Next, we conducted the wound healing assay and transwell migration assay to evaluate the impact of TNFRSF11B interference on LGG cell migration. The wound healing assay showed that interference of TNFRSF11B significantly inhibited the migration ability of CHG5 and HS683 cells at 24 h (*p* < 0.01,* p* < 0.0001, Fig. 9D, E), 36 h (*p* < 0.0001, *p* < 0.0001, Fig. 9D, E) and 48 h (*p* < 0.0001, *p* < 0.001, Fig. 9D, E). The transwell migration assay also showed that interference with TNFRSF11B significantly reduced the migratory numbers of CHG5 (*p* < 0.0001, Fig. 9F, G) and HS683 (*p* < 0.0001, Fig. 9F, G) cells. Taken together, our findings indicate that interference with TNFRSF11B expression inhibits both the proliferation and migration of LGG cells.

## Discussion

LGG is a common aggressive tumor in the CNS and has the potential to evolve into the most malignant glioblastoma. LGG is not completely curable by surgical resection due to the unique immune infiltration mechanism and the presence of glioma stem cells in the CNS [45–47]. Currently, postoperative adjuvant radiotherapy and temozolomide chemotherapy remain the main treatment strategies. However, recent studies have shown that identifying key biomarkers is essential to improving the survival of LGG patients [6, 7]. Researchers have focused on cuprotosis, a newly discovered mode of cell death, which offers a new perspective for improving cancer treatment [48, 49]. In this study, we comprehensively explored the role and prognostic significance of cuprotosis in LGG. Through the analysis of 18 cuprotosis genes, the first finding was that there were two distinct clusters of LGG patients with different clinical outcomes, immune cell infiltration, and biological functions. The second finding was the development of a prognostic signature based on 6 differential genes between the clusters, which could aid in predicting the prognosis of LGG patients. Additionally, the third finding showed that changes in the TME and TMB were associated with risk scores, indicating a potential mechanism for the relationship between cuprotosis and immune response. Lastly, interfering with the expression of the signature gene TNFRSF11B was found to inhibit LGG cell proliferation and migration, providing potential targets for therapeutic interventions.

Firstly, we found that LGG samples could be split into two clusters according to the expression of 18 cuprotosis genes [8, 17–19]. Cuprotosis cluster A showed a better prognosis than cuprotosis cluster B. Surprisingly, different immune cell infiltration and biological functions were found between two cuprotosis clusters. Previous studies have emphasized the significant role of TME in tumor malignancy and response to immunotherapy [50–52]. For example, epithelial and stromal cells were involved in tumor growth, malignant progression, and therapeutic resistance [53, 54]. Infiltrating immune cells such as macrophages and lymphocytes also exhibited tumor-promoting features [55, 56]. A study specifically focusing on LGG and TME correlation showed that high TME scores predicted a poor prognosis for LGG patients [57]. This was also consistent with our findings in this study, where we observed a higher degree of immune cell infiltration and a poorer prognosis in cuprotosis cluster B. Interestingly, we also found that immune and tumor-related pathways were predominantly enriched in cuprotosis cluster B. These findings suggested that cuprotosis genes may influence the prognosis of LGG patients by regulating immune cell infiltration via immune- and tumor-associated pathways.

In light of the significant differences between the two cuprotosis clusters, we delved deeper into the genetic makeup of both clusters. We identified 1370 DEGs between cuprotosis clusters and conducted GO functional enrichment analysis that highlighted the DEGs' importance in leukocyte-mediated immunity, positive regulation of cell adhesion and cytokine production (BP). Leukocyte-mediated immunity, which is a vital response mechanism of the immune system that involves the participation of various types of leukocytes such as neutrophils, macrophages, and lymphocytes. Neutrophils are the most abundant type of leukocyte in humans, and their infiltration degree was significantly correlated with glioma grade [58]. The presence of cell adhesion molecules such as adhesion molecule on glia (AMOG) and neural cell adhesion molecule L1 (L1CAM) plays a vital role in regulating the growth and progression of gliomas, where increased L1CAM expression and decreased AMOG expression are correlated with the degree of malignancy [59]. Cytokines in the glioma microenvironment also play a crucial role in aiding the progression of the disease. They can be segregated into chemokines, invasive factors, angiogenic factors, immunosuppressive factors, and glioma-associated microglia and macrophages (GAM) polarizing factors, which promote GAMs to transform into the tumor-promoting phenotype, the M2 phenotype [60]. Other factors, such as hepatocyte growth factor, play a significant role in the mitogenesis and mobility of gliomas [61]. Additionally, IL-6 activates the STAT3 signaling cascade, leading to increased VEGF expression, which promotes tumor angiogenesis and growth [62]. Overall, these pathways provide valuable insights into the immune infiltration and the prognosis differences between the cuprotosis clusters.

To further investigate the association between the DEGs and the prognosis, LGG patients were categorized into geneCluster A and geneCluster B based on the DEGs via consensus clustering analysis. Similar to the cuprotosis cluster, LGG patients in geneCluster A had a comparatively better prognosis than those in geneCluster B. On the contrary, cuprotosis cluster B corresponded to geneCluster B, a poorer prognosis, a higher clinical grade, and older populations, indicating that cuprotosis could be used as a distinguishing factor for LGG patients.

However, owing to the heterogeneity in the expression of cuprotosis genes, the aforementioned clustering analysis could not provide individual prognosis assessments for LGG patients. Given this, a prognostic signature relying on cuprotosis-related DEGs was established for accurate prognosis prediction in LGG patients. The signature emerged as a reliable predictor for the prognosis of LGG patients through internal and external validation. To further equip the clinicians with prognosis estimates, a nomogram was created to predict the 1-, 3-, and 5-year OS of LGG patients according to each patient’s risk score and clinical characteristics. Furthermore, our research also uncovered new findings concerning the correlation between the risk score and immune cell infiltration. Results indicated that the risk score was positively correlated to the infiltration of M0 macrophages, and CD8 T cells while being negatively related to the infiltration of activated NK cells, eosinophils, monocytes, and activated mast cells. These results were consistent with previous research that discovered PDGF-DD-activated NK cells predicted a better prognosis for LGG patients [63]. The presence of immune checkpoints, including PD-1, PD-L1, CTLA4, LAG3, TIM-3, and GAL9, can be linked to immunotherapy responsiveness [32–35]. Our study revealed that LGG patients displaying higher risk scores had higher expressions of immune checkpoints, which suggests that high-risk patients might be more likely to benefit from immunotherapy. Therefore, the use of cuprotosis-related prognostic signature holds promise for predicting the clinical effectiveness of immunotherapy in LGG patients.

In our study, we identified six signature genes, namely TNFRSF11B, METTL7B, SSTR2, OXTR, CDKN2C, and H19 which have been extensively studied in the past and are known to play a significant role in cancer. TNFRSF11B, also known as osteoprotegerin, is a protein that regulates bone homeostasis and has been shown to play a role in cancer development [64–66]. TNFRSF11B activation of the Wnt/β-catenin signaling pathway has been found to promote the progression of gastric cancer [65]. Similarly, METTL7B is critical for cell cycle progression and tumorigenesis in non-small cell lung cancer [67]. SSTR2 is a receptor that is often dysregulated in different types of tumors, including neuroendocrine tumors, breast cancer, lung cancer, and prostate cancer [68–74]. The activation of SSTR2 inhibits the proliferation of tumor cells primarily through growth arrests [75]. The increased level of OXTR mRNA may indicate a poor prognosis for patients with colon adenocarcinoma [76]. CDKN2C expression is linked to TMB and TME, suggesting its potential use as a prognostic marker for immunotherapy [77]. Finally, H19 is upregulated in multiple types of cancer, including gastric cancer, colorectal cancer, breast cancer, ovarian cancer, and glioma, and also correlates with poor prognosis in these cancers [78–82]. While previous studies have identified the important roles these genes play in cancer progression, our study provides additional insight into their specific association with cuprotosis in LGG. Additionally, we found that TNFRSF11B plays an oncogenic role in LGG, and inhibiting TNFRSF11B can effectively suppress the proliferation and migration of LGG cells. Our study provides a guide for future in-depth research studies and targeted therapies in LGG based on cuprotosis.

## Conclusions

Overall, this study highlights the relevance of cuprotosis in LGG, and the identified prognostic signature may aid in predicting clinical outcomes, evaluating immunotherapy response, and guiding the development of new therapies in LGG patients.

### Supplementary Information

Below is the link to the electronic supplementary material.

Supplementary file1 (TIF 2047 KB)
Correlation analysis of risk score with the expression of immune checkpoints. (A-F) Correlation analysis of PD-1, PD-L1, CTLA4, LAG3,TIM-3, and GAL9 expression with the risk score.

## Data Availability

All data generated and analyzed in this study are included in this article and its supplementary files. The data analyzed during the current study are available in The Cancer Genome Atlas (TCGA, https://portal.gdc.cancer.gov/) database and Chinese Glioma Genome Atlas (CGGA, http://www.cgga.org.cn/) database.

## Abbreviations

AMOG: Adhesion molecule on glia
AUC: The area under the curve
BP: Biological process
CC: Cellular component
CNS: Central nervous system
CGGA: Chinese Glioma Genome Atlas
CNV: Copy number variants
CDF: Cumulative distribution function
DEGs: Differentially expressed genes
DLD: Dihydrolipoamide dehydrogenase
GAM: Glioma-associated microglia and macrophage
GSVA: Gene set variation analysis
GO: Gene Ontology
KM: Kaplan–Meier
KEGG: Kyoto Encyclopedia of Genes and Genomes
LASSO: Least absolute shrinkage and selection operator
L1CAM: Neural cell adhesion molecule L1
LGG: Low-grade glioma
MF: Molecular function
OS: Overall survival
PCA: Principal components analysis
PDHA1: Pyruvate dehydrogenase E1α subunit
ssGSEA: Single sample gene set analysis
TCGA: The Cancer Genome Atlas
TME: Tumor microenvironment
TMB: Tumor mutation burden
IDH1: Isocitrate dehydrogenase 1
