# Supplementary figures and images for: Cuprotosis clusters predict prognosis and immunotherapy response in low-grade glioma
Source: Apoptosis. 2023 Sep 15;29(1-2):169–90. doi: 10.1007/s10495-023-01880-y (PMC10830610; doi:10.1007/s10495-023-01880-y)

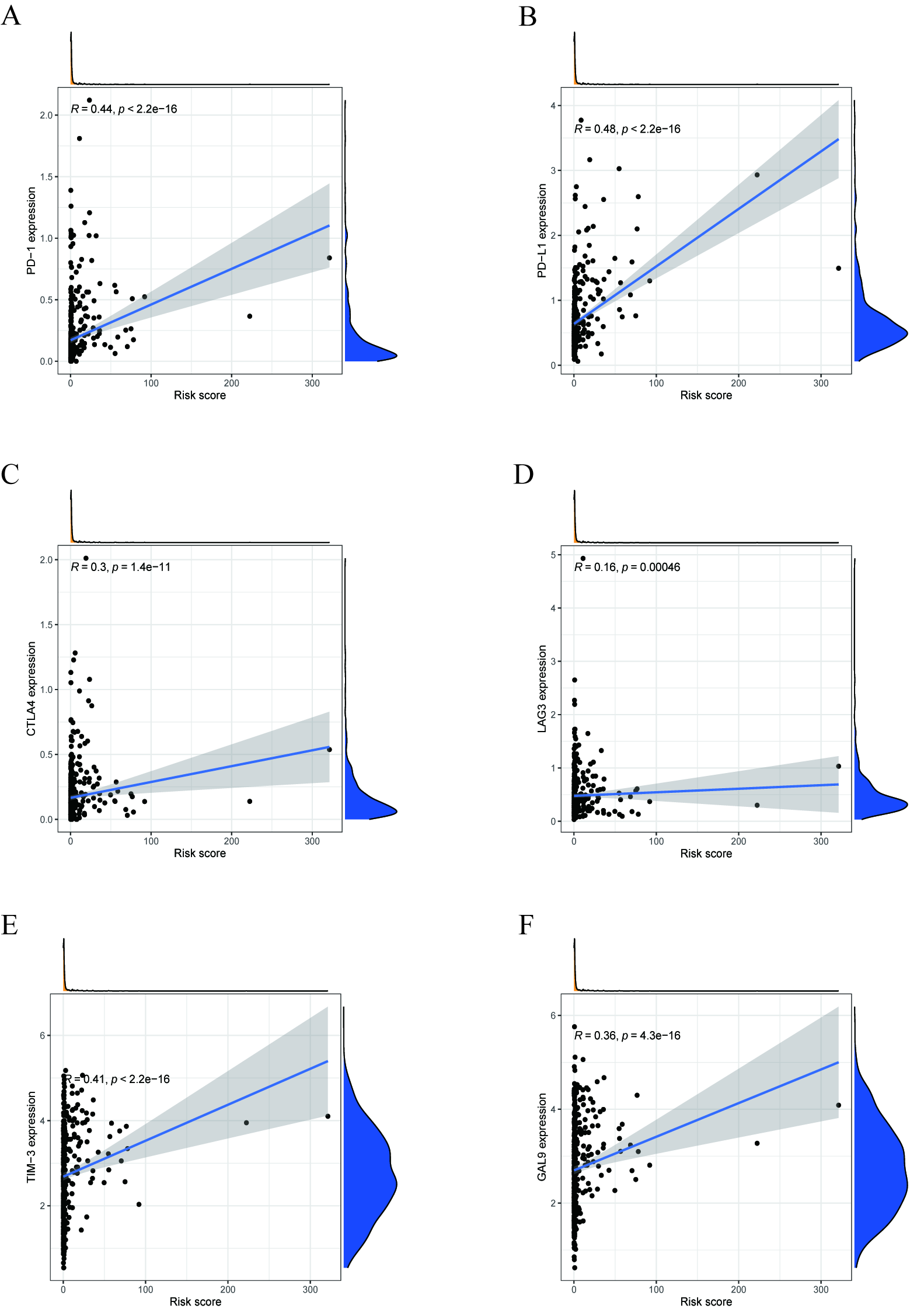

Supplement: Supplementary file 1 — Supplementary file1 (TIF 2047 KB) Correlation analysis of risk score with the expression of immune checkpoints. (A-F) Correlation analysis of PD-1, PD-L1, CTLA4, LAG3,TIM-3, and GAL9 expression with the risk score. [file 10495_2023_1880_MOESM1_ESM.tif]
